# Supplementary material for: Development of Proniosome Gel Formulation for CHIKV Infection
Source: Pharmaceutics. 2024 Jul 26;16(8):994. doi: 10.3390/pharmaceutics16080994 (PMC11360264; doi:10.3390/pharmaceutics16080994)
Supplement: Supplementary file 1 [file pharmaceutics-16-00994-s001.zip › pharmaceutics-3096193-supplementary.pdf]

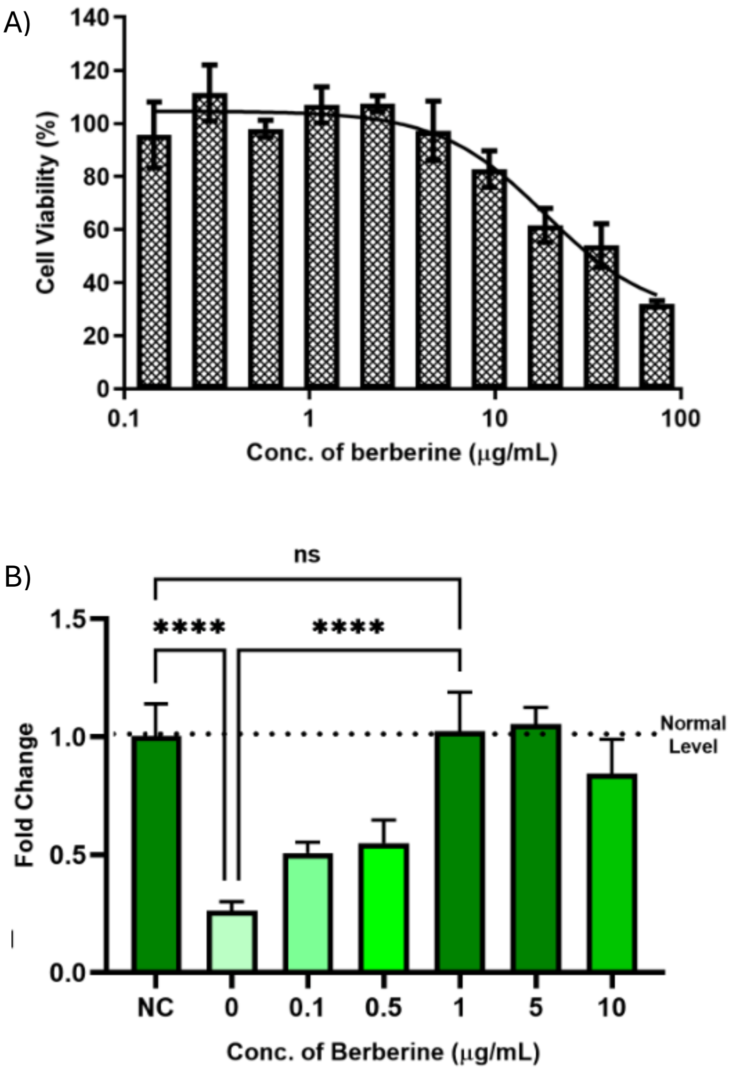

Supplementary Figure S1. (**A**) Cytotoxic profile of berberine on inflamed chondrocytes, induced by IL-1β and TNF-α, and keratinocytes. (**B**) Fold change in nitric oxide (NO) and sGAG synthesis production when inflamed chondrocytes, induced by IL-1β and TNF-α, were treated with berberine
